# Gender Differences, Motor Skills and Physical Fitness Heterogeneity in Adults with Down’s Syndrome

**DOI:** 10.3390/jcm12041367

**Published:** 2023-02-08

**Authors:** Sandro Covain, Sébastien Baillieul, Thai Duy Nguyen, Michel Guinot, Stéphane Doutreleau, Véronique-Aurélie Bricout

**Affiliations:** Inserm U1300, CHU Grenoble Alpes, HP2, Université Grenoble Alpes, 38000 Grenoble, France

**Keywords:** Down’s syndrome, physical activity, physical fitness, motor skills, cluster analysis

## Abstract

*Background*—Adults with Down’s syndrome (DS) present lower physical fitness associated with heightened sedentary behaviors and motor skills impairments. Their etiologies and determinants seem to be heterogeneous. This study aims to evaluate physical fitness in adults with DS and to identify specific physical fitness profiles depending on gender and physical activity levels. *Methods*—Forty adults with DS (16 women, 24 men, 29.7 ± 7.5 years) performed six tests from the EUROFIT Battery and Motor Assessment Battery for Children (MAB-C). Their maximal aerobic capacity was assessed using an incremental treadmill test to assess (VO_2peak_). Ecological, physical activity, and sedentary levels were evaluated subjectively (Global Physical Activity Questionnaire) and objectively using an Actigraph GT9X^®^ accelerometer over a seven-day period. *Results*—VO_2peak_ and isometric strength were significantly lower for women (*p* < 0.01), whereas men had significantly lower flexibility than women (*p* < 0.05). Using a principal component analysis and an agglomerative hierarchical analysis, we identified three clusters. Cluster 1 (n = 14; 50% men; Body Mass index = 28.3 ± 4.3) was characterized by significantly poorer physical fitness variables (VO_2peak_ (*p* < 0.01), strength (*p* < 0.01) and balance (*p* < 0.05)) compared to Clusters 2 and 3. Cluster 2 (n = 19; 58% men; Body Mass index = 22.9 ± 2.0) and Cluster 3 (n = 19; 58% men; BMI = 22.9 ± 1.9) were characterized by subjects with comparable physical fitness profiles, except for the balance capacities, which were significantly lower in Cluster 3 (*p* < 0.05). *Conclusions*—DS subjects exhibited high heterogeneity in terms of physical fitness, PA, and sedentary levels, with a significant gender effect. The present findings are important to identify subjects at higher risk of sedentary behaviors and impaired motor capacities to develop personalized PA programs.

## 1. Introduction

Down’s syndrome (DS) is a genetic disorder resulting from the presence of all or part of a third copy of the 21st chromosome, with a prevalence of 10.4/1000 [1]. This syndrome is characterized by a wide spectrum of clinical signs affecting the musculoskeletal, neurological, and cardiovascular systems. More precisely, DS’s phenotype is associated with low height, overweight or obesity, cognitive impairments, endocrine co-morbidities, cardiovascular developmental abnormalities, dysautonomia, a high prevalence of sleep apnea, and osteo-articular abnormalities [2,3]. Early diagnosis eases targeted care, including stimulation by education or physical activity (PA). PA triggers well-described large benefits regarding life expectancy and quality of life [2] and physical fitness [4]. If the benefits of regular PA in the general population have been demonstrated, there are currently no specific PA guidelines for people with DS. Fox et al. reported that children with DS do not reach the PA levels recommended in the international guidelines (an average of 60 min of moderate to vigorous PA daily), with the intensity of PA levels declining as children enter adolescence [5,6]. Adults with DS do not reach the international PA recommendations for adults too, with a low daily PA level and a prolonged duration of sedentary behavior [7,8]. Specifically, in this population, sedentary behavior appeared higher than previously reported values in the general population. In a recent review, Agiovlasitis et al. [7] showed that the multivariate sedentary behavior correlates were primarily familial (higher socio-economic status, higher mother’s education and work status) and environmental factors (such as TV, walkable community). Numerous musculoskeletal, cardiovascular, and biological characteristics of this genetic syndrome, such as atlanto-axial instability or lumbar spinal stenosis, muscle hypotonicity, hyperlaxity, lower muscular strength, and cardiovascular fitness, may limit their daily activity, including PA. These physiological characteristics of the population with Down’s syndrome result in potential limitations and restrictions to physical exercise. However, if these specificities can be a barrier to a competitive sports practice, they are not limiting factors for a leisure practice. Social, environmental and familial factors are more influential to promote or limit a physically lifestyle in adult with intellectual deficiencies than physiological one [9,10].

Down’s syndrome’s related motor impairments may lead to high levels of a sedentary lifestyle and decreased physical fitness, characterized by a lower aerobic capacity in adults when compared to healthy subjects [11,12,13,14]. Low levels of motivation for any PA [11,15] related to a negative perception of motor capacities and practice environment may contribute to sedentary lifestyle behaviors, the lack of PA engagement, and, finally, impaired physical fitness [5,9,10,16,17]. On the other hand, PA interventions may also be helpful in improving impaired motor skills. Thus, in children with DS, PA programs have been proposed to improve motor skills impairments linked with motor developmental deficiencies [5,13]. In adults with DS, these studies remain scarce. There are no recommendations for the population with intellectual disabilities with or without DS, and therefore the recommendations are the same for adults without disabilities insofar as possible [6]. The few studies available were mainly focused on aerobic capacity assessment [11,12,13,14,18]. Collectively, these studies confirmed that adults with DS have low physical fitness levels and are at increased risk for several co-occurring diseases (such as obesity and diabetes), which are known to benefit from increased PA [2,13,19,20]. The determinants of PA levels and motor capacities related to physical fitness in adults with DS are poorly studied.

In 2022, a study using the Motor Assessment Battery for Children (MAB-C) reported results evaluating the motor capacities of adults with DS as a function of PA level [21]. The authors demonstrated a positive effect of PA level on manual dexterity, static and dynamic balance, and general motor coordination but lacked a proper evaluation of the effects of age and gender. If the authors highlighted the need to implement PA programs to enhance motor and cognitive skills in DS adults, they did not identify whether a specific subgroup should be specifically targeted by such interventions. However, DS’s phenotypes and individual needs are highly heterogeneous. It is thus essential to identify the motor capacities of individuals with DS to develop personalized PA interventions.

The aim of this exploratory study was to describe physical fitness profiles in adults with DS. Second, we applied statistical classification methods to highlight the gender and PA levels related to physical fitness in this population.

## 2. Materials and Methods

Prospective observational study conducted at the Grenoble Alpes University Hospital (France). The study was approved by the National Ethics Committee Sud Méditerranée (France; n°2017-AOI914-49) and was conducted according to the principles of the Declaration of Helsinki. Each participant received oral and written information and signed a consent form prior to participation in the study (Clinical trial registration number: NCT03445962).

### 2.1. Participant Characteristics

Forty participants with DS (n = 24 (60%) men, 29.7 ± 7.5 years; 18 to 46 years) were recruited through employment support services or local associations. To be included, participants had to be 18 or older and to present no contra-indication to PA participation. All medical conditions that could alter the response to exercise were retained as non-inclusion criteria (asthma or other respiratory infection, diabetes, leukemia, psychiatric disorders, osteo-articular pathology). The level of intellectual functioning was mild to moderate. the participants did not have trouble comprehending instructions or questions., All participants lived with their parents. None had epilepsy or neuro-anatomical disorders (such as atlanto-axial instability or lumbar spinal stenosis). Population description is presented in Table 1.

### 2.2. Experimental Protocol

Each participant underwent a medical examination to assess the absence of contra-indication and to collect anthropometric parameters. The Global Physical Activity Questionnaire (GPAQ [22]) was completed by the participants and their parents. GPAQ was translated into Met-minutes per week (Metabolic equivalent of task) based on the intensity and duration of activity. The higher the value, the more active the subject.

With reference to our previous work [23] and according to the definition given by the American College of Sports Medicine [24], (that precise that health-related physical fitness includes at least the evaluation of body composition, aerobic components (VO_2_), muscular strength and flexibility), motor capacities were evaluated with different tests from the EUROFIT Physical Fitness Test Battery [25], and from Movement Assessment Battery for Children (MAB-C; [26]).

Each participant was tested individually by the same experienced therapist in the same order (Eurofit, MAB-C and VO_2_ measure). The total of the experimental protocol lasted for one morning.

### 2.3. Measures

EUROFIT tests

EUROFIT tests evaluated 4 components of the motor capacities:

- Flexibility. The sit and reach flexibility test is a common measure of flexibility of the lower back and hamstring muscles. The participant was sitting on the floor, legs stretched out, and was asked to bend the trunk and reach forward as far as possible. Any measure beyond the toes line was positive, and any measure below was negative. A higher score (expressed in centimeters) indicated better flexibility.
- Explosive strength. The participant jumps as far as possible during a broad jump test. The horizontal distance achieved is measured in centimeters
- Isometric strength. A handgrip test (Sensel Measurements force sensor, France) recorded the maximum voluntary force of preferential hand squeezing. A higher score (expressed in Newton) indicated better isometric strength.
- Static balance. Time held on one leg is timed in seconds.

For all these tests, the higher the score, the better the performance.

MAB-C battery

Two tests from the MAB-C battery were used to assess balance abilities.

- Static balance (both feet aligned on a beam: time (in seconds) held in balance and which determines a score between 0 and 4.
- Dynamic balance (number of steps backward without imbalance on a line drawn on the ground and which determines a score between 0 and 4).

The higher the scores, the greater the balance impairments.

VO_2peak_ measure. Each participant performed an incremental aerobic treadmill test (Gymrol Super 2500, Andrézieux, France) to determine the maximum oxygen consumption (VO_2peak_, in mL·kg^−1^·min^−1^ and L·min^−1^). After familiarization (3 km/h for 2 min), the speed and the slope of the treadmill were increased alternatively every minute by 1 km/h or 2%, respectively, until exhaustion. During this incremental test, gas exchanges were measured continuously using an ergospirometer (Medisoft^®^ Ergocard, CPX pro, Medisoft S.A. Dinant–Sorinnes (Belgium)). The predicted VO_2peak_ value was calculated using Wasserman’s formula [27], which then allowed the calculation of %VO_2predicted_. A %VO_2predicted_ value below 80% determines an effort limitation.

Actimetry. PA levels were recorded using the Actigraph GT9X^®^ (Actigraph Corp, TSP diffusion, Poissy, France) accelerometer over a seven-day period, including weekend days. Participants wore the monitor at the waist for all activities except showering or swimming. A daily diary was completed in parallel to record accelerometer-free periods (i.e., shower), subsequently removed from the analysis. Raw accelerometer counts were downloaded using Actilife 6^®^ software (version 7.0), and the time spent in sedentary behavior or in PA was calculated with Freedson’s algorithm adapted for the calculation of PA times for adults [28].

### 2.4. Statistical Analysis

Results are presented as mean ± standard deviations. First, characterization of the population (n = 40) was carried out after checking the normality of the variables using the Shapiro–Wilk test and the homogeneity of the variable using Levene’s test. Independent samples t-tests or Mann–Whitney tests were used to compare clinical, questionnaire, PA evaluations, and actimetry variables between men (n = 24) and women (n = 16). Second, a classification model aimed to identify physical fitness characteristics of Down’s syndrome. A principal component analysis (PCA) and an ascendant hierarchical cluster analysis (AHCA) were performed on data (Venables). The PCA was supplied with the normalized version of the original predictors. Here, normalization and centralization of the data by the feature scaling method were first applied [29]. In this part of our study, we had n = 40 observations (40 adults with DS) and 26 predictors, so a parsimonious model by the Elastic net algorithm was used to select the most relevant variables from 26 variables [30]. Variables selected were gender (A), VO_2peak_ (B), flexibility (C), isometric strength (D), explosive strength (E), dynamic balance (F), BMI (G), and static balance factors (H). Our two static balance variables have been factorized to respect the application rule for PCA: no more than 5 observations per prediction. The significance level was set at *p* < 0.05. Statistics were performed using Jamovi (version 2.6) and R (version 4.2.1).

## 3. Results

Characteristics of the population

Descriptive demographic data and physical fitness variables related to gender are presented in Table 1. Men were taller and heavier compared to women (*p* < 0.001, and *p* < 0.01, respectively; Table 1). VO_2peak_ and isometric strength results were significantly lower for women (*p* < 0.01; Table 1), whereas men had significantly lower flexibility than women (*p* < 0.05; Table 1). There were no gender-related differences for actimetry variables or GPAQ scores.

Classification of the population by gender and PA level

**Figure 1:**
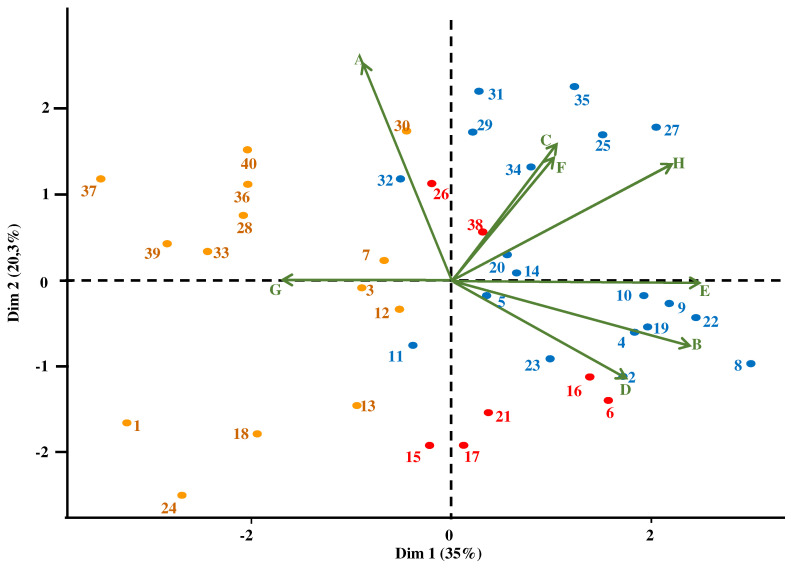
Principal component analysis biplot. Participants with DS are represented from 1 to 24 for men and 25 to 40 for women. Each point represents a subject and the color its membership in the cluster. On this graph, two diametrically opposed vectors are negatively correlated, two vectors forming an acute angle are positively correlated, two vectors forming a 90° angle are independent of each other. The greater the distance between the vectors, the closer their relationship is to the significant threshold. A subject near a vector reveals a high score for this variable, a subject diametrically opposed to a vector reveals a low score for this value. A: gender, B: VO_2peak_, C: Flexibility, D: isometric strength, E: explosive strength, F: dynamic balance, G: BMI, H: static balance factor.

Relationships were further explored using a clustering approach, which allowed us to classify the participants based on the PA and physical fitness variables. PCA results showed that 55.3% of the variance was explained by eight significant variables grouped in the first two dimensions (35% and 20.3%, respectively, eigenvalue ≥ 1), which represents a satisfactory outcome. This analysis ranked each individual on a factor map, using different colors to indicate the membership of specific items from different clusters. Regarding variable distribution (Figure 1), we observed that women participants were exclusively grouped in the top half, while men participants were grouped in the lower half of the biplot (Figure 1), indicating a clear gender effect.

**Figure 2:**
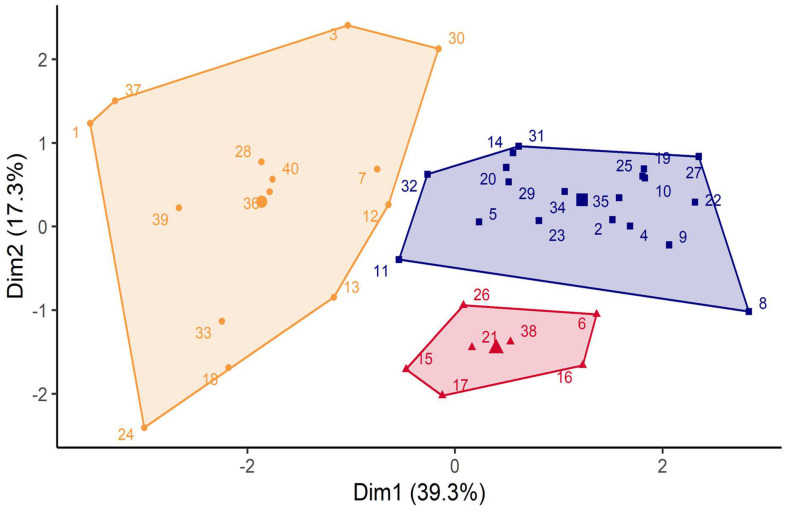
Factor map obtained by agglomerative hierarchical cluster analysis. Cluster 1 (orange) included 7 males and 7 females with the highest age and BMI and the lowest physical fitness and physical activity level. They were characterized by an effort limitation (%VO_2predicted_ < 77%); Cluster 2 (blue, 12 males and 7 females) and Cluster 3 (red, 5 males and 2 females) were characterized by normal BMI and very good VO_2peak_ values. Participants in Cluster 2 had the highest static and dynamic balance score. Participants in Cluster 3 had the highest flexibility, explosive and isometric strength.

Factor map representation allowed us to specifically localize the position of one subject within its cluster and to compare it with neighboring clusters (Figure 2). The least active adults were those with lower physical fitness and with very low daily PA levels (Cluster 1).

Descriptive demographic data and physical fitness variables by the cluster’s PA level (related to actimetry evaluation) are presented in Table 2. Cluster 1 included 14 participants (7 men/50%), Cluster 2 included 19 participants (12 men/58%), and Cluster 3 included 7 participants (5 men/40%). In Cluster 1, subjects were significantly older than subjects in Clusters 2 and 3 (34.4 ± 7.5 vs. 27.1 ± 6.2 and 25.7 ± 5.6 yrs, respectively, *p* < 0.05) and had a significantly higher BMI than subjects in Clusters 2 and 3 (28.3 ± 4.3 vs. 22.9 ± 2.0 and 22.9 ± 1.9 kg/m^2^; *p* < 0.001 and *p* < 0.01, respectively). Cluster 1 is characterized by poor physical fitness profiles (VO_2peak_ (*p* < 0.01)) and an exercise limitation (77%). Explosive strength (*p* < 0.01) and balance (*p* < 0.05) were significantly lower in Cluster 1 compared to Clusters 2 and 3, with a lower GPAQ score (*p* < 0.05; *p* < 0.01, respectively).

Cluster 2 and 3 are characterized by subjects with comparable physical fitness profiles, except for the balance abilities, which are significantly lower in Cluster 3 compared to Cluster 2 (static balance: 9″71 ± 5″92 vs. 17″50 ± 3″08, *p* < 0.05 and dynamic balance: score: 4.86 ± 0.97 vs. 14.00 ± 2.16, *p* < 0.001). GPAQ scores were significantly higher in Cluster 3 than in Cluster 2 (score: 3443 ± 2529 vs. 3060 ± 1836, *p* < 0.05).

## 4. Discussion

This study aimed to describe physical fitness profiles in a comprehensive group of adults with DS. In this work, we showed a clear gender effect regarding physical fitness in adults with DS. Only a few studies to date have reported comprehensive motor and cardiorespiratory capacities assessments and their relation with gender or PA levels in adults with DS [31,32,33,34,35,36,37].

### 4.1. Gender Differences

In our study, we highlighted specific differences in physical fitness between men and women, differences that are the same as those observed in the general population. As shown previously in numerous studies, the physical fitness of participants with DS was lower than in typically developed adults. Indeed, we expressed the VO_2peak_ measured as the percent of the predicted VO_2_, which was established in the US general population (Table 1). The results currently showed that the cardiorespiratory fitness of participants with DS is lower than typically developed adults.

However, our work reported these results according to gender, which had not yet been performed in the studies published to date, which grouped the subjects into a single group without dissociating them according to gender.

Women had a significantly higher flexibility test score than men, while men had a significantly better isometric strength score, suggesting greater muscle strength. The increase in flexibility in female adults may be related to gender differences in hormonal or anatomical changes that have occurred during puberty. It is well shown that women are more flexible than men [38]. The results of this study in DS are consistent with those in the general population. Moreover, flexibility is conditioned by muscle mass and stiffness: the greater the muscle mass, the higher the stiffness.

Isometric strength was significantly lower for women, whereas men had significantly lower flexibility than women. Some studies have assessed these strength and flexibility capacities in adults with DS and the impact of a training program, but none have reported a specific gender effect on these capacities. This may be due either because the study involved only men or because the number of women included in these studies was too small to conclude. In 2022, Islam et al. [34], in a descriptive cohort study including 468 participants with DS (2–84 years; 56% men) and using an interview with a structured questionnaire and clinical examination, reported gender-related differences but the authors did not specify the nature of the motor capacity that differed between men and women. This result is consistent with Winter et al. [39], who showed in children with DS no statistically significant difference between males and females on all 44 gross motor skills assessments. These discrepancies between studies may be explained by the age-related impairment in motor capacities and the associated decline in PA levels in DS [13]. This phenomenon has been previously described by Oppewal and Hilgenkamp [40], who highlighted the need to maintain a sufficient level of PA to limit the decline in the physical condition of aging adults with DS.

We further showed that absolute and relative VO_2peak_ values were significantly lower for women (both *p* < 0.001. If many studies were focused on the impaired aerobic capacity in DS [13,31,32,41,42], none specifically focused on gender differences. The low value of cardiovascular fitness in DS is often explained as a result of obesity [13,32,33,43,44,45], but in our cohort, participants were not obese on the mean, and there was no difference in mean BMI values between men and women.

### 4.2. Physical Activity Levels and Their Potential Determinants

The second important outcome of this work was the multimodal PA level assessment and the identification of 3 different clusters of physical fitness profiles and PA levels. Cluster 1 covered participants with a low VO_2peak_ value and significantly impaired motor capacities compared to participants in the two other clusters. In Cluster 1, participants exhibited the lowest PA levels, as assessed by the PA questionnaire (GPAQ, subjective measure) or by actimetry (objective measure). The observed PA levels were far below the current recommendations for PA levels associated with general health benefits for adults (Adults should do at least 150–300 min of moderate-intensity aerobic physical activity; or at least 75–150 min of vigorous-intensity aerobic physical activity; or an equivalent combination of moderate- and vigorous-intensity activity throughout the week, for substantial health benefits) [6,46]. The time spent in sedentary behaviors was high, further increasing the risk of gaining weight, obesity, and cardiometabolic co-morbidities [46]. Nevertheless, in agreement with the WHO, the recommendations for sedentary behavior per day < 480 min max. The subjects of our study were close to this value. It is mainly the time spent in moderate and vigorous activities that were not sufficient. This low PA level may contribute to the low physical fitness in this population, and it has been found that it results in functional activity impairments [5]. Our results are in agreement with those of previous studies confirming that adults with DS are less active and tend to engage in more light-intensity PA than MVPA compared to adults without DS [13,47] but also than their peers with intellectual disabilities without DS [48]. This observation is consistent throughout the developmental life of individuals with DS, being reported similarly in children [36,49], in adolescents [17] as well as in adults [13,47].

We also observed a clear age effect. In Cluster 1, participants were significantly older than in Clusters 2 and 3 (mean age 34.4 ± 7.5 vs. 27.1 ± 6.2 and 25.7 ± 5.6 yrs, respectively). According to previous studies, individuals with DS present poor static and dynamic balance compared to control participants [50,51,52] and poor strength [13,17]. While there is a growing body of studies evaluating the PA program’s benefits in children with DS to prevent these impairments, there have been relatively few published reports on the effects of strength or balance training programs in adults with DS. Nevertheless, the relationship between muscle strength, balance, and aerobic capacity is repeatedly used to characterize physical health and the early onset of age-related diseases [13,31,32,41,42]. Balance capacities are essential as they improve motor coordination [53] and determine the subject’s engagement in leisure and social activities [53]. Muscle weakness and poor standing balance capacities are associated with an increased risk of falling [54]. Adapted PA may improve these and all other motor abilities [53]. In our study, Cluster 1 was composed of individuals with the more impaired motor capacities associated with the lowest PA levels, for whom it would be important to propose adapted PA programs, targeting specifically the physical fitness impairments. Regardless of the determinants of insufficient PA levels, improving motor capacities and aerobic capacity is essential. The development of specific PA programs should be encouraged to prevent or maintain the physical fitness components, to slow down the onset of muscle weakness and to decondition secondary to sedentary lifestyles, and to promote healthier ways of living.

Thus, in 2002, Carmeli et al. [55] demonstrated the beneficial effects of a walking program on strength and balance (3 days/week; 30 min minimum; 15 weeks). Subsequently, many other programs have demonstrated the benefits of PA on the physical fitness of adults with DS, either with swimming or walking programs (3 days/week; 30 min minimum, 8–15 weeks) [56,57], combined aerobic-resistance training (3 days/week; 30 min minimum, 8–15 weeks) [18], sprint activities (3 days/week; 30 min minimum, 15 weeks) [58] and basketball (3 or 4 days/week; 45 min minimum, 8–24 weeks) [53,59]. These studies all used training programs with this design: 3 days a week, 30 min minimum, for a total duration of 6 to 24 weeks, with mainly endurance activities, and often based on collective games (Basketball) to improve compliance and participation. Results of these studies showed PA benefits on motor capacities, physical fitness, cognitive function, social skills, and psychological well-being in adults with DS. However, all of these studies reported one-off effects of a PA program proposed over a limited period (a few weeks to a few months, in small adult population) but it would be useful to have a follow-up cohort over several years, with different ages and gender subgroups.

There is, therefore, a huge need to build interventional cohorts of PA assessment in DS and to better identify the determinants of PA limitations, such as the strengthening of these gender differences that may help the development of targeted interventions to improve physical fitness and function. Hence, recommendations for multidisciplinary management of Down’s syndrome must include regular physical activity [60,61,62,63] in an appropriate environment (recreational practice, transport facilities, adapted structures, and adapted physical activities).

The latest recommendations published in 2020 by the Workgroup of the Global Down’s syndrome Foundation Medical Care [64] were not very indicative in the field of PA and did not provide specific guidelines for the adult population with DS and should be updated in that sense. There is not a sufficient level of evidence to publish recommendations for T21. Recommendations for the general population can be proposed, and to encourage PA in this population, it will be necessary to play on the environment, on the “influences and barriers” [6,9,10,65].

The stake is to maintain in adulthood the motor and cardiorespiratory capacities developed during childhood. The challenge of promoting physical interventions in adulthood in DS is to reduce the slope of physical weakening in a population that is less fitted than adults typically developed, and so it needs specific adaptations. Nevertheless, intensive sports practice provides significant improvement in motor abilities, such as in typically developed athletes. Thus, adults with DS are able to train, perform, and take part in national and international Paralympic games considering their disability. It is likely that early interventions during childhood contribute to making future sportsmen [62].

Our study had some limitations and strengths. Our study was cross-sectional and monocentric, with a limited population, limiting the external validity of the study. To increase confidence in our observations, including control subjects may help to better understand the link between physical fitness impairments and PA levels. The strengths of our study were the use of a large panel of PA assessments, including objective methods by actimetry coupled with a subjective method. The application of original exploratory statistics was interesting but should be reinforced by models allowing the investigation of the causal link between the different variables.

## 5. Conclusions

We identified a specific cluster characterized by poor physical fitness associated with a low and insufficient level of PA. PA is a key factor in improving health and motor capacity performances in adults with DS. Personalized approaches should be developed in order to improve physical fitness. Future studies should strengthen the characterization of the described physical fitness and PA profiles, their evolution through time as well as their impact on social and cardiovascular health and well-being of individuals with DS.

## Figures and Tables

**Table 1:** Participants’ anthropometric characteristics, physical fitness and PA assessment related to gender.

		Men (n = 24)	Women (n = 16)
Demographic	Age (years)	29.8 ± 6.2	28.8 ± 9.2
	Height (cm)	161 ± 5	146 ± 5 ***
	Weight (kg)	63.98 ± 9.1	53.9 ± 9.6 **
	BMI (kg/m^2^)	24.7 ± 3.6	25.0 ± 4.4
Physical fitness	VO_2peak_ (mLO_2_·kg^−1^·min^−1^)	36.1 ± 7.7	29.5 ± 7.9 **
	VO_2peak_ (L·min^−1^)	2.27 ± 0.45	1.52 ± 0.38 ***
	VO_2predicted_ (mLO_2_·kg^−1^·min^−1^)	39.45 ± 2.32	32.60 ± 4.64 ***
	%VO_2predicted_	91.4 ± 18.3	90.8 ± 21.0
	Flexibility (cm)	26.3 ± 10.2	32.0 ± 9.2 *
	Explosive strength (cm)	100.9 ± 36.5	89.3 ± 32.6
	Isometric strength (N)	280.4 ± 98.6	182.8 ± 72.7 **
	Static balance (s)	11″96 ± 6″49	13″58 ± 5″93
	Dynamic balance (score)	10.50 ± 5.63	11.38 ± 4.76
Physical activity	GPAQ	2940 ± 2173	2019 ± 1311
	Sedentary behavior (min)	469.5 ± 98.1	483.8 ± 77.5
	MVPA (min)	29.37 ± 23.02	21.92 ± 10.23
	Energy expenditure	269.4 ± 99.1	225.6 ± 109.6
	Number of steps	6301 ± 1716	6125 ± 1841

Values are means ± SD. BMI: Body mass index was calculated (BMI: body weight in kg/height in m^2^). VO_2predicted_ see methods. Tests details: see Method Section 2. Low values of flexibility represent a low performance. Strength: the higher the value, the higher the performance. GPAQ: global physical activity questionnaire. MVPA: moderate to vigorous physical activity. Sedentary behavior, MVPA, Energy expenditure, Number of steps: data obtained by actimetry. Significantly different from men * *p* < 0.05; ** *p* < 0.01; *** *p* < 0.001.

**Table 2:** Participants’ anthropometric characteristics, physical fitness and PA assessment related to physical activity level.

		Cluster 1 (n = 14)	Cluster 2 (n = 19)	Cluster 3 (n = 7)
Demographic	Age (years)	34.4 ± 7.5	27.1 ± 6.2 *	25.7 ± 5.6 *
	Height (cm)	1.53 ± 0.08	1.56 ± 0.08	1.56 ± 0.08
	Weight (kg)	65.9 ± 11.4	56.9 ± 9.1	56.3 ± 7.2
	BMI (kg/m^2^)	28.3 ± 4.3	22.9 ± 2.0 ***	22.9 ± 1.9 **
Physical fitness	VO_2peak_ (mLO_2_·kg^−1^min^−1^)	25.41 ± 4.37	37.69 ± 6.90 **	38.19 ± 5.89 ***
	VO_2theo_ (L·min^−1^)	1.85 ± 0.58	1.95 ± 0.54	2.03 ± 0.48
	VO_2predicted_ (mLO_2_·kg^−1^·min^−1^)	33.44 ± 5.31	38.32 ± 3.73 *	38.88 ± 2.84
	%VO_2predicted_	77.0 ± 13.8	98.9 ± 18.6 **	98.4 ± 14.8 *
	Flexibility (cm)	25.01 ± 12.55	30.41 ± 8.77	30.71 ± 7.05
	Explosive strength (cm)	68.85 ± 31.26	108.92 ± 29.66 **	116.86 ± 21.63 **
	Isometric strength (N)	186.26 ± 92.55	269.35 ± 101.84	275.57 ± 71.62
	Static balance (s)	7″41 ± 4″38	17″50 ± 3″08 ***	9″71 ± 5″92 $
	Dynamic balance (score)	9.57 ± 6.05	14.00 ± 2.16 *	4.86 ± 2.97 $$$
Physical activity	GPAQ	1473 ± 1134	3060 ± 1836 *	3443 ± 2529 **$
	Sedentary behavior (min)	478 ± 101	485 ± 75	442 ± 108
	MVPA (min)	20.27 ± 13.68	29.32 ± 22.43	30.67 ± 18.23
	Energy expenditure	252.7 ± 98.8	237.1 ± 112.3	290.0 ± 96.7
	Number of steps	5716 ± 1596	6161 ± 1553	7450 ± 2166

Values are means ± SD. VO_2predicted_ see methods_;_ GPAQ: Global physical activity questionnaire. Sedentary behavior, MVPA, Energy expenditure, Number of steps: data obtained by actimetry. Significantly different from Cluster 1: * *p* < 0.05, ** *p* < 0.01, *** *p* < 0.001; from Cluster 2: $ *p* < 0.05, $$$ *p* < 0.001.

## Data Availability

The data presented in this study are available on request from the corresponding author. The data are not publicly available due to privacy and ethical restrictions.
